# Unveiling Time in Dose-Response Models to Infer Host Susceptibility to Pathogens

**DOI:** 10.1371/journal.pcbi.1003773

**Published:** 2014-08-14

**Authors:** Delphine Pessoa, Caetano Souto-Maior, Erida Gjini, Joao S. Lopes, Bruno Ceña, Cláudia T. Codeço, M. Gabriela M. Gomes

**Affiliations:** 1 Instituto Gulbenkian de Ciência, Oeiras, Portugal; 2 Programa de Computação Científica, Fundação Oswaldo Cruz, Rio de Janeiro, Brazil; ETH Zurich, Switzerland

## Abstract

The biological effects of interventions to control infectious diseases typically depend on the intensity of pathogen challenge. As much as the levels of natural pathogen circulation vary over time and geographical location, the development of invariant efficacy measures is of major importance, even if only indirectly inferrable. Here a method is introduced to assess host susceptibility to pathogens, and applied to a detailed dataset generated by challenging groups of insect hosts (*Drosophila melanogaster*) with a range of pathogen (*Drosophila* C Virus) doses and recording survival over time. The experiment was replicated for flies carrying the *Wolbachia* symbiont, which is known to reduce host susceptibility to viral infections. The entire dataset is fitted by a novel quantitative framework that significantly extends classical methods for microbial risk assessment and provides accurate distributions of symbiont-induced protection. More generally, our data-driven modeling procedure provides novel insights for study design and analyses to assess interventions.

## Author Summary

While control options for plant, animal, and human pathogens are emerging rapidly, reliable assessment of the effect of interventions in biological systems presents many challenges. A major question is how to connect laboratory experiments and measurements with the relevant process in natural settings, where hosts are subject to pathogen exposures that vary in time and geographical location. With this aim, measures of protection that are invariant under varying exposure intensity need to be developed and integrated with mathematical models. In this article, we introduce a method to assess host susceptibility to pathogens, and apply it to survival of *Drosophila melanogaster* challenged with different doses of *Drosophila* C virus. By replicating the procedure in groups of flies that carry the symbiont *Wolbachia*, we are able to estimate how the viral protection induced by this intracellular bacterium is distributed in the host population. Our results disentangle host infection status from observed mortality, accounting naturally for time since exposure. The multiple-dose design proposed challenges traditional study designs to assess interventions.

## Introduction

Hosts exposed to disease-causing agents respond in accordance to the challenge dose. Therefore dose-response curves contain information about disease processes that can be extracted by suitable analytic frameworks. Early examples concerning microbial risk assessment include counting lesions caused by tobacco mosaic virus on plant leaves [1], as well as human responders to experimental challenge with polio viruses [2], *Vibrio cholerae* [3] and *Streptococcus pneumoniae* [4], for escalating challenge doses. Dose-response models have been in use for analyses and extrapolation of experimental datasets [5].

Models that account for the sigmoidal shape in log-linear scale of the typical dose-response curve have been derived mechanistically, based on the assumption that each individual pathogen has a probability of infection independent of others, the so-called independent action hypothesis [6]. This results in a one-parameter exponential-function model [7]. The frequent observation of shallower-than-exponential, or overdispersed, relationships has then prompted the implementation of heterogeneity in the probability of infection of individual hosts [8]–[10].

In the 1960s, Furumoto and Mickey [9] developed a dose-response model that could accommodate both shallow and steep increases in the response by considering the probability of infection of individual hosts described by a Beta-distribution. Although a mechanistic justification for this specific distribution has not been given, the model has been widely applied in microbial risk assessment due to its ability to outperform the simple exponential model [5].

Susceptibility distributions other than Beta have also been considered and are more commonly used in frailty models adopted in survival analysis [11], where the data consist of survivor counts over time in host groups that are constantly subject to a hazard [12], [13]. These frailty models appeared in the 1980s and have since been adapted to infection hazards, where surviving signifies remaining uninfected [14]–[16]. While most informative when the exposure is continued or repeated over time, these formalisms would be inadequate for estimating distributions of susceptibility to infection from instantaneous challenge protocols.

The importance of accounting for time between challenge and observable toxicity responses to pathogens or other agents has been recognized. Recent models in ecotoxicology [17], [18], consider explicit kinetics within exposed organisms. Also in microbial risk analysis, previous studies [19], [20] have included time postinoculation as an additional parameter in classic dose-response models, although using an approach that conceptually allows for a different susceptibility distribution at each time point. Here we present a schema to infer a distribution of host susceptibilities to infection that holds consistently across dose and time. We introduce an experimental design and inference framework that enables such inferences by analyzing simultaneously a collection of survival curves, each representing a different challenge dose. The resulting Beta distributions are compared against those obtained by classic dose-response models based on single day measurements.

Recent evidence for symbiotic interactions that reduce host susceptibility to pathogens has stimulated the development of quantitative frameworks to assess the levels of individual and population protection attributable to specific symbionts. The intracellular bacterium *Wolbachia*, found among many arthropod species including *Drosophila melanogaster*, is one such symbiont [21], [22]. To analyze the protection conferred by *Wolbachia* to *D. melanogaster*, we apply our inference framework simultaneously to two sets of time-dependent dose-response data: in one set the flies carry the symbiont bacterium *Wolbachia* (Wolb^+^); while in the other they do not (Wolb^−^). In this instance we extract the Beta distribution that best describes individual protection attributable to *Wolbachia*, as well as population statistics valid across entire dose ranges.

## Methods

### Survival data

We used virus free *D. melanogaster* lines with DrosDel w^1118^ background, with or without the endogenous *Wolbachia* strain wMelCS [21], [23], [24]. Flies were reared in standard food at 25°C. To assure that potential for heterogeneities are minimized by the experimental procedure, we used fifty 3–6 days old adult males per group, 10 per replicate and 5 replicates. To study the response to viral infection, we anesthetized with CO_2_ and pricked flies with different doses of *Drosophila C virus* (*D*CV). We used tenfold serial dilutions – from 10^10^ TCID_50_/ml to 10^4^ TCID_50_/ml – in Tris-HCl buffer, pH 7.5. Controls were pricked with buffer solution only. We used the pricking protocol described in [24], produced and titrated virus as in [21]. After pricking, we kept flies at 18°C and checked daily survival until day 80 and twice a week until the end of the experiment. Food was changed every 5 days. We summarized the data in 16 dose-response curves (8 per group, including control) from day 0 after treatment until day 139 (Dataset S1).

### Dose-response model

Starting from established models, we refine the occurrence of mortality from infection, i.e. the *response*, as a function of the concentration of infectious units given to hosts, i.e. the *dose*. We present a step-by-step derivation of descriptions that integrate dimensions that are usually treated separately as well as the motivations for doing so.

Assuming independent action of infectious units, each unit has probability *p* of causing an infection, while for *d* infectious units infection occurs with a probability described by 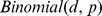. Given further considerations about the distribution of infectious units in a homogeneous solution (see [9] for a complete derivation of the expression), the number of units causing infection can be described by a Poisson distribution, resulting in the exponential dose-response model [7], that describes the probability of infection in a host challenged with pathogen dose *d*:
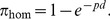
(1)This most basic formulation is hereafter referred to as the homogeneous dose-response model.

Furumoto and Mickey [9] expanded this formulation by allowing the probability of infection to be described by a parametric distribution, specifically the Beta distribution. To facilitate normalization across datasets, here we maintain the probability *p* fixed across individual hosts (as in [25]), and introduce a multiplicative parameter, the susceptibility factor 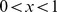, to describe any natural or induced effect that decreases susceptibility. We assume that susceptibility to infection is Beta-distributed so as to describe the variation of susceptibility in the host population. Thus, we obtain the probability 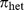 that a host contracts infection as
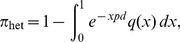
(2)where 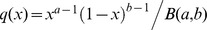 and *B* is the Beta function. We refer to this formulation as the heterogeneous dose-response model.

At last we introduce a small parameter *ε* to account for a small probability of ineffective challenge, such that 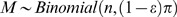 is the random variable representing the number of infected hosts, in a group of *n* hosts challenged with a given dose. Assuming that an ineffectively challenged host behaves like a control host with regard to death rates, the probability that *m* hosts are dead a number of days after challenge is then
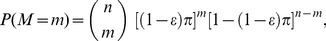
(3)where 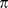 is either 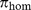 (1) or 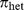 (2) depending on which dose-response model is adopted.

The parameters to be estimated for this dose-response model are the maximum probability of infection per infectious unit (*p*), the shape parameters for the Beta distribution that describes the susceptibility factor (*a*, *b*), and the probability of ineffective challenge (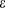).

These models require a choice of how many days post-challenge cumulative mortality should be measured, which is difficult to establish for host-pathogen systems where times to death from infection or other causes overlap significantly. To overcome this difficulty, we develop a model that integrates an explicit representation of time to death with the dose-response process for infection just described. It should, however, be noted that time is introduced with the main purpose of enabling the use of survival curves to obtain robust estimates for probabilities of infection given different challenge intensities and consistently infer susceptibility to infection. From this perspective, parameters defined from now on should be regarded as auxiliary and will be implemented as simply as possible.

### Time-dependent model for control group

We first consider a survival model for a control group of flies pricked with buffer solution only (no *D*CV), subject to two hazards: 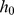, an age-dependent death hazard rate; and 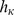, a background age-independent death hazard rate. The overall death hazard rate for uninfected hosts is therefore
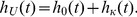
(4)Denoting 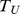 the random variable representing time to death of control hosts, we have
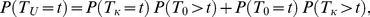
(5)where 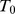 and 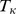 are the times to death from 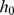 and 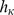, respectively. Their corresponding distributions are assumed to be 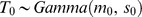 and 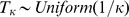, where 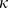 is the background mortality rate, 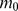 is the mean time to death, and 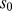 is the shape parameter for the Gamma distribution of day of death from aging.

### Time-dependent dose-response model

Hosts challenged with pathogen can become infected or remain uninfected and this infection status is hidden. If uninfected, they are subject to the age-dependent hazard rate that affects control hosts, 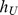; if infected, they are subject to an infection hazard rate, 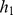, and the age-independent background mortality. Thus the overall hazard rate of infected hosts is
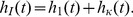
(6)Now let 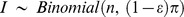 be the random variable representing the number of hosts infected by challenge with a given pathogen dose. Then the probability that *i* hosts are infected after *n* hosts were challenged is
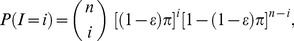
(7)where 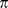 is either 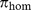 (1) or 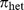 (2) depending on which dose-response model is adopted.

Let *T* be the random variable representing the time to death of hosts challenged by a given pathogen dose. The probability density of observing a death event at time *t* given that *i* hosts are infected is
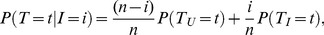
(8)where 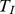 denotes the distribution of time to death of infected hosts, given by
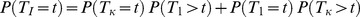
(9)and 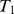 is the distribution of times to death from the infection hazard rate 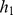. This distribution is assumed to follow 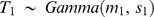, where 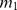 is the mean time to death of infected hosts, and 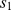 is the shape parameter for the Gamma distribution of day of death from infection.

In setting the priors for parameter estimation we note that background mortality is small and therefore 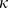 is kept small by setting 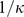 to be much greater than the last day of the experiment. To enforce that deaths due to infection occur earlier than deaths due to aging, we constrain the mean time to infection death to be lower than old-age death, i.e. 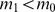, and the probability of dying before the end of the study to be greater for infected hosts, i.e. 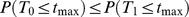, where 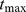 is the last day of the experiment.

To construct the likelihood to be maximized by the parameter estimation procedure, we let 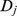 be the random variable denoting the day fly 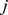 died and 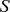 the random number of survivors up to 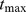. Then the likelihood of observing the actual number of survivors 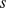 and the times of death 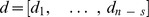, for a given dose is
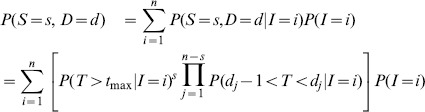
(10)Since the observations for each dose are independent, taking the product of the likelihoods over the different doses yields the global expression for the likelihood of the entire dataset.

In this time-dependent dose-response model, the parameters to be estimated are the maximum probability of infection per infectious unit (*p*) used for normalization purposes, the Beta distribution shape parameters to describe variation in susceptibility factor (*a,b*), the parameters that control death due to aging (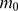, 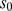), infection (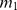, 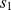), and background mortality (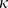), as well as probability of ineffective challenge (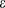). Parameters 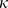 and 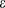 are typically small and were introduced to improve performance of the likelihood.

### Parameter estimation

Model parameters were estimated using Markov chain Monte Carlo sampling implemented with the PyMC package [26] (code available from [27]). The prior distributions considered are listed in Table 1. Initial values were chosen so as to start with a non-zero likelihood. Using Metropolis-Hastings algorithm, we ran two separate chains for 252,000 iterations. The first 27,000 iterations were discarded. The recording interval was set to 250 so that the autocorrelation between samples was negligible. Convergence was assessed by inspection of the trace plots. All analyses were performed on the pooled samples from the two replicate chains.

**Table 1:** Model parameters and their corresponding prior distributions.

Symbol	Meaning	Prior
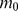	Mean time to death from aging	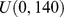
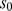	Shape of the Gamma distribution for death from aging	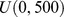
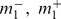	Mean time to death from infection (for Wolb^−^ and Wolb^+^, respectively)	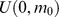
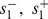	Shape of the Gamma distribution for death from infection (for Wolb^−^ and Wolb^+^)	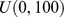
*p*	Per viral particle probability of causing infection	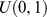
*a*, *b*	Shape parameters of the Beta distribution for the susceptibility to infection of Wolb^+^	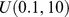
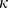	Background mortality rate, from causes other infection or aging	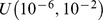
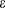	Probability of ineffective challenge	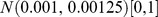

*U*(*x*,*y*) is a Uniform distribution from *x* to *y*. *N*(*x*,*y*)[*w*,*z*] is a normal distribution with mean *x* and standard deviation *y* truncated so its values are always between *w* and *z*.

## Results

**Figure 1:**
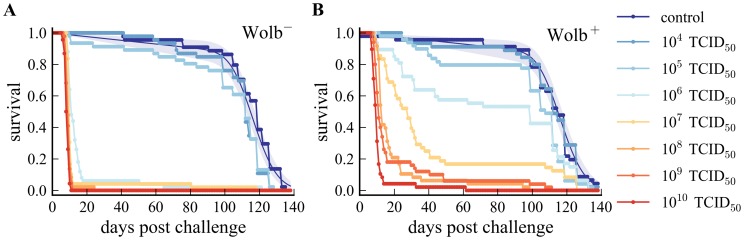
Survival curves for Wolb^−^ (A) and Wolb^+^ (B) groups of *D. melanogaster*. Dots represent experimental data. Dark blue curves show the model fit to the survival of control flies. Shaded areas represents 95% CI (credible intervals).

Groups of *Wolbachia*-negative (Wolb^−^) and positive (Wolb^+^) *D. melanogaster* flies were challenged with a range of *D*CV doses and survival curves were traced as shown in Figure 1. This dataset was analyzed by applying the models introduced in Methods.

### Susceptibility distribution from selected day mortality

**Figure 2:**
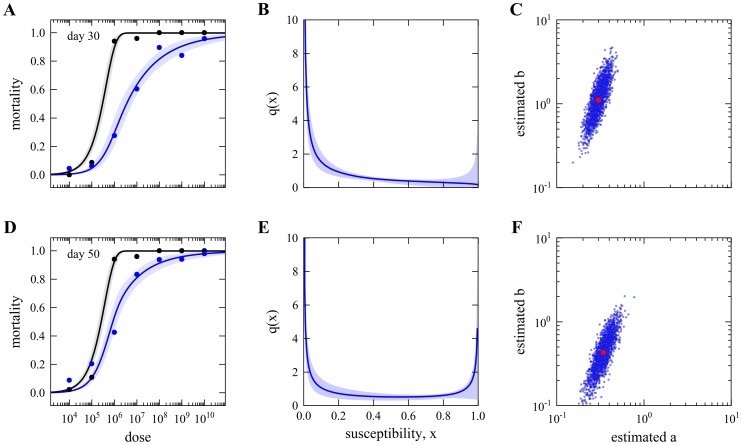
Dose-response curves and susceptibility distributions inferred from mortality measurements 30 and 50 days post-challenge. Dose-responses models adopted here are the standard formulations (1–3). A,D, Curves represent the fitted dose-response model to mortality on selected day post-challenge (dots), for Wolb^−^ (black) and Wolb^+^ (blue). Shaded areas represent the 95% CI. B,E, Distribution of susceptibility to infection in Wolb^+^. The posterior median distribution is the curve and the shaded area is the 95% CI. C,F, Posterior samples of the Beta-distribution shape parameters describing Wolb^+^ susceptibility in blue. Red dot mark the median of the respective distributions. The homogeneous model was adopted for Wolb^−^.

**Figure 3:**
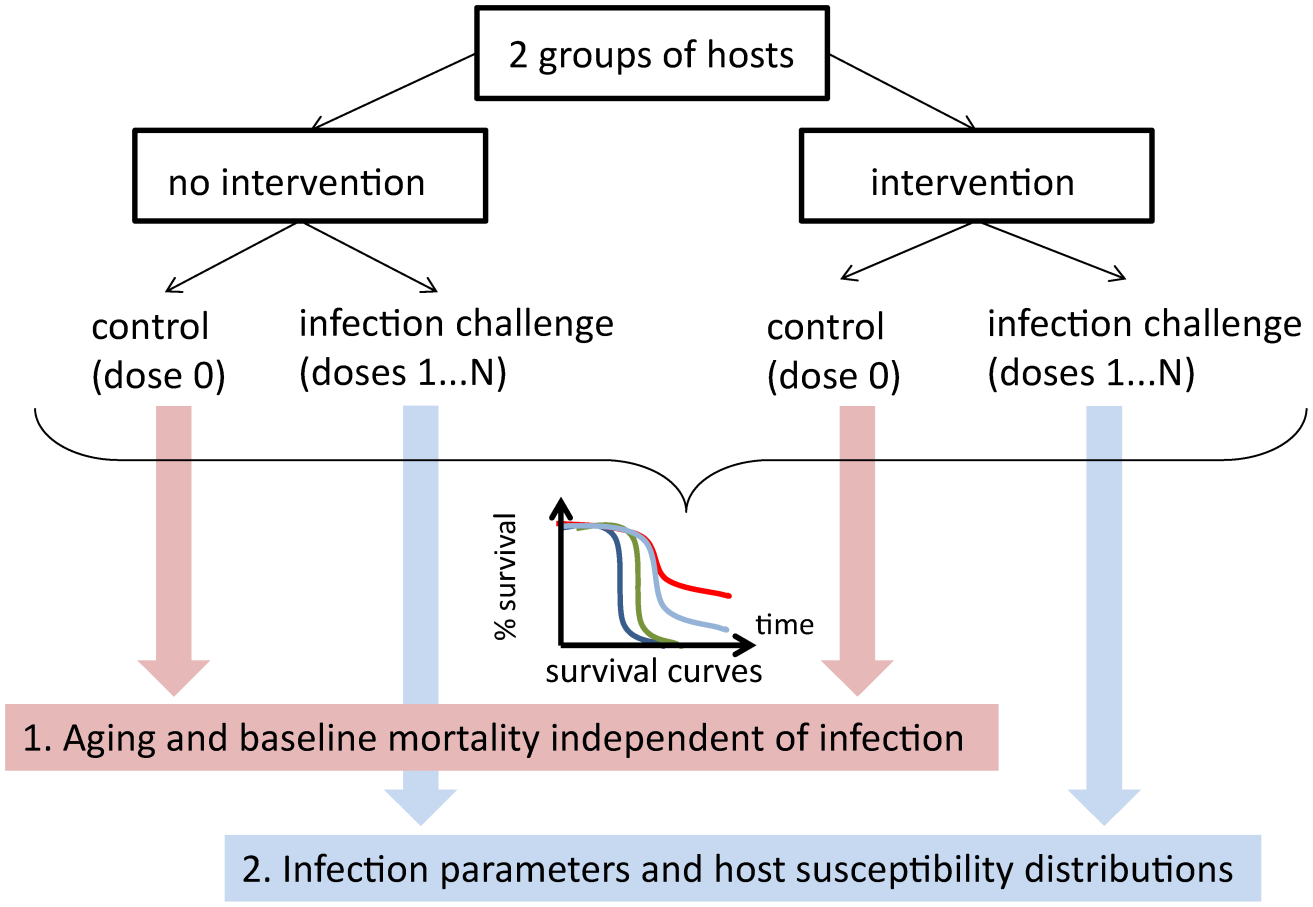
Schematic illustration of the proposed experimental design and inference procedure.

To emphasize the importance of day selection to infer distributions of susceptibility to infection by classic dose-response models [5] we have applied these procedures to mortality data observed by two specific days (30 and 50). Parameter estimates from these models are listed in Table 2. The model fits to the mortality data at the selected days are shown in Figure 2, as well as the associated distribution of Wolb^+^ susceptibilities and the posterior samples for the Beta distribution shape parameters. For simplicity we have adopted the homogeneous model for Wolb^−^ and focus on comparing susceptibility distributions of Wolb^+^ inferred at different days. Mean protection conferred by *Wolbachia* in this illustration is estimated as 79% and 56%, based on mortality measurements at day 30 and 50, respectively. Moreover, the distributions have fundamentally different shapes, with the appearance of a high susceptibility group as time progresses. This sensitivity to the day by which mortality data are collected is a concern that raises the need to disentangle infection status from the associated time-dependent mortality. In the following sections, infection and mortality are estimated explicitly using the integrated time-dependent model described in Methods. The procedure is illustrated in Figure 3.

**Table 2:** Estimated parameters by applying dose-response models to selected day mortality.

Mortality data	Parameter	Median	95% HPD^a^
30 dpc^b^	*p*	2.33 10^−6^	[1.67 10^−6^, 3.13 10^−6^]
	*a*	0.30	[0.21, 0.41]
	*b*	1.10	[0.29, 2.53]
	*ε*	1.78 10^−3^	[4.90 10^−4^, 3.49 10^−3^]
50 dpc^b^	*p*	2.65 10^−6^	[1.82 10^−6^, 3.47 10^−6^]
	*a*	0.34	[0.24, 0.51]
	*b*	0.42	[0.12, 0.93]
	*ε*	1.83 10^−3^	[3.60 10^−4^, 3.32 10^−3^]

a High posterior density interval.

b Days post-challenge.

### Aging and background mortality

Control curves from Wolb^−^ and Wolb^+^ flies pricked with buffer solution (no *D*CV) were compared with the Kaplan-Meier method using the log-rank test and no significant difference was found (with a p-value of 0.47). By fitting the uninfected time-dependent model (4–6) to the control survival curves (Figure 1) we estimated the parameters describing aging (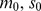) and background (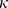) mortality (Table 3).

**Table 3:** Estimated parameters governing time to death from causes other than *D*CV infection.

Parameter	Median	95% HPD
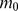	117.18	[114.99, 119.84]
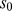	118.93	[80.19, 166.15]
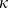	1.14 10^−3^	[5.36 10^−4^, 1.96 10^−3^]

### Susceptibility distribution from survival curves

**Figure 4:**
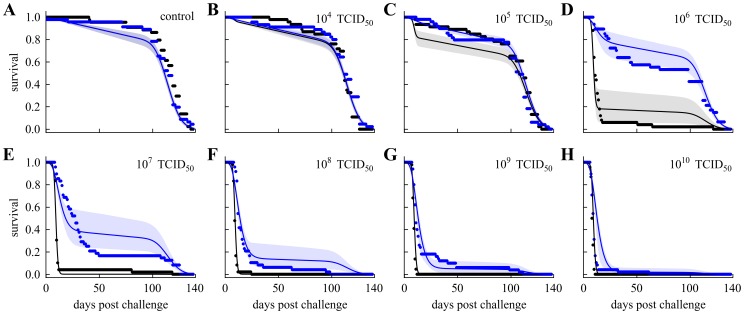
Fit of time-dependent dose-response model to survival curves. Black and blue dots are the observed proportions surviving over time for Wolb^−^ and Wolb^+^ groups, respectively. The curve is the fitted mean posterior survival over time and the shaded area is the 95% CI. Fifty flies per group were pricked with: A, buffer solution (shown for comparison but not used on this analysis); and B, 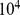; C, 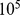; D, 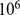; E, 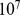; F, 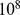; G, 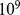; H, 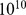 TCID_50_ *D*CV.

For each group of flies (Wolb^−^ and Wolb^+^), the time-dependent dose-response model constructed in Methods was fitted simultaneously to the entire dataset of survival curves (one for each *D*CV challenge dose), fixing across doses the distribution of times to death from infection (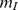, 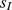) and aging (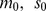), while estimating the susceptibility parameters (*p*, *a*, *b*) that govern the dependence of response on challenge dose according to the adopted dose-response model. The estimated parameter values are listed in Table 4. The deviance information criterion (DIC) [28] favored the homogeneous model for the Wolb^−^ group and the heterogeneous model for Wolb^+^ (Text S1). Mean time to death from infection is 9 and 14 days in the Wolb^−^ and Wolb^+^ groups, respectively. The variance in time to death from infection is lower for Wolb^−^, with a standard deviation of 2 days, compared to 6 days in the Wolb^+^. Figure 4 compares fitted with observed survival curves.

**Table 4:** Parameters governing estimated number infected per dose of *D*CV challenge and time to death from infection using time-dependent dose-response models described in Methods.

Parameter	Median	95% HPD
*p*	1.73 10^−6^	[9.58 10^−7^, 2.67 10^−6^]
*a*	0.47	[0.25, 0.85]
*b*	3.21	[0.34, 8.40]
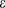	1.89 10^−3^	[4.55 10^−4^, 3.40 10^−3^]
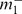	9.34	[9.10, 9.58]
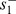	35.79	[26.60, 47.05]
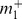	13.79	[11.31, 14.94]
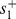	5.59	[4.70, 11.12]
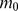	115.20	[113.94, 116.45]
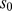	140.39	[116.80, 166.97]
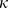	2.15 10^−3^	[1.65 10^−3^, 2.71 10^−3^]

Parameters with superscripts ^−^ and ^+^ relate to Wolb^−^ and Wolb^+^ groups, respectively.

**Figure 5:**
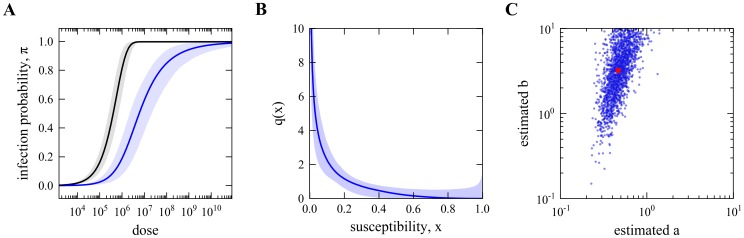
Dose-response curves and susceptibility distributions inferred from survival curves. A, Curves represent the estimated dose-response relationships from fitting the model described in Methods to survival over time, for Wolb^−^ (black) and Wolb^+^ (blue). Shaded areas represent the 95% CI. B, Distribution of susceptibility to infection in Wolb^+^. The posterior median distribution is the curve and the shaded area is the 95% CI. C, Posterior samples of the Beta-distribution shape parameters describing Wolb^+^ susceptibility in blue. Red dot marks the median of distribution.

The fitted dose-response curves that result from this analysis are shown in Figure 5A, while the inferred distribution of Wolb^+^ susceptibilities normalized by the Wolb^−^ measure is displayed in Figure 5B and the corresponding posterior distribution of the Beta shape parameters is in Figure 5C. Given the homogeneity in the Wolb^−^ group, the distribution of susceptibility in Wolb^+^ provides a direct indication of how antiviral protection conferred by *Wolbachia* is distributed among its carriers. Typically defined as 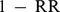, where RR is the risk reduction attributed to the susceptibility modifier (*Wolbachia* in this case), we determine the mean protection conferred by the symbiont to its host as 85% (with a 95% HPD of 60–93%).

### Comparison with selected day mortality

**Figure 6:**
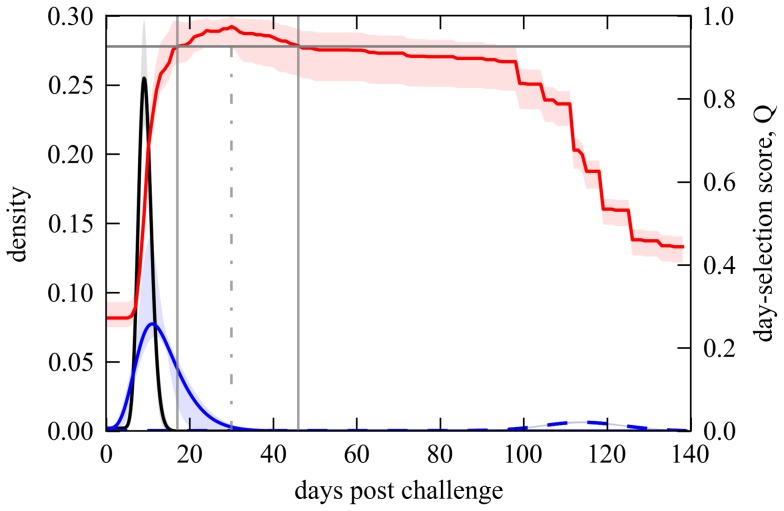
Selection of optimal days to collect mortality measurements for traditional dose-response models. The red line traces a score for how well mortality at any given day represents infection estimated by the time-dependent model (refer to axis on the right). The score is given by 
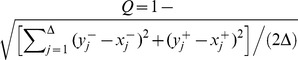
, where Δ denotes the number of doses in the dataset, 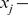 (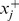) represents the proportion infected in the Wolb^−^ (Wolb^+^) group subject to *D*CV dose *j*, and 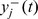 (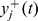) the observed mortality proportion over time in the Wolb^−^ (Wolb^+^) group subject to *D*CV dose *j*. Gray vertical lines mark the optimal day to measure mortality for dose-response models (day 30, dash-dotted line) and the limits of the acceptable range (days 17 and 46). Dashed lines represent the Gamma distributions that describe old-age mortality, and black (blue) full curves refer to the Gamma distributions that describe infection-induced mortality in Wolb^−^ (Wolb^+^) (refer to axis on the left). Curves are the mean posterior probabilities and shaded areas represent the 95% CI.

To assess the best possible performance of classic methods [5] in the inference of susceptibility distributions (for Wolb^+^ in the case) we must have previously reduced the set of survival curves to a set of effectively infected proportions - one entry per challenge dose. To search for a range of days in which absolute mortality might provide an approximate indication of infection, we compare the estimated proportions effectively infected by each challenge dose with the mortality proportion measured at each day. Using a normalized Euclidean distance between these two measures, a day-selection score is provided by the red curve in Figure 6. We identify day 30 as optimal and 17–46 as the interval of days in which the score is at least 95% of the optimal. Reassuringly, the optimal day appears to coincide with the saturation of infection-induced mortality (see position of vertical dash-dotted gray line in relation to the Gamma distributions). We now recall Figure 2 and Table 2 for the inferences based on day 30 mortality data to confirm that classic dose-response models can in principle infer susceptibility distributions that are consistent with those obtained under our extended model (Figure 5). A major issue, however, is that results are sensitive to a day-selection criterion that relies on having previously carried out the entire procedure. The appearance of a high susceptibility group in distributions inferred at later days are an artifact due to the accumulation of background mortality that should be factored out. These results highlight the importance of adequately representing the time dimension in the analysis.

## Discussion

Dose-response models have become standard quantitative frameworks in microbial risk assessment. Less recognized is their ability to estimate host trait distributions. Here we illustrate the concept by extracting host susceptibility distributions from mortality measured as a function of pathogen challenge dose, but similar procedures can be developed for measures of infection or infectiousness (instead of mortality), and can be made a function of other environmental variables such as temperature or humidity (instead of dose). Understanding how to detach host trait distributions from environmental variables is crucial for the formulation of measures that can be transported between laboratory and natural conditions [29], [30].

We address this problem with an experimental design and inference framework that enables the estimation of distributions of host susceptibility to infection by analyzing simultaneously a collection of survival curves, each representing a different challenge dose (Figure 3). The procedure is illustrated on a specifically collected dataset where two distinct groups of hosts (*D. melanogaster*) were experimentally challenged by viruses (*D*CV): one group consists of isogenic flies where no significant variability in susceptibility to infection is found; and another with the same genetic background but now carrying the symbiont bacterium *Wolbachia* known to reduce susceptibility to *D*CV [21], [22].

Our inferences indicate that *Wolbachia* confers on average 85% *D*CV protection to *D. melanogaster* under the specified laboratory conditions, and suggest significant variability in this effect. This variance in susceptibility is induced by the symbiont, since model selection criteria did not support heterogeneity in the susceptibility of flies not carrying *Wolbachia*. Since the Drosophila and *Wolbachia* populations used in this study are isogenic, the heterogeneity in susceptibility of *Wolbachia*-carrying flies uncovered here indicates variation in the host-microorganism interaction that lacks a genetic basis. A simple hypothesis is that variance in *Wolbachia* levels at the individual host level leads to variance in resistance to viruses. Although several lines of evidence support this hypothesis [31]–[34], further experiments are required to discriminate whether heterogeneity in resistance is directly linked to variance in *Wolbachia* levels or, alternatively, a result of another environmental/physiological variance that is only expressed in the presence of *Wolbachia*.

Previous estimates of protection were based on survival analysis or viral titres in a dose-specific manner [21], [22], [24]. To our knowledge, the experimental design and analysis presented here provides the first estimation of protection in way that is detached from challenge dose. Future developments might consider: estimation of alternative distributions to compare with the shapes suggested by the Beta family; extension of the adopted experimental design to measure responses other that mortality; and move towards host populations and environmental conditions that are closer to natural systems.

The parameters estimated here should not be seen as isolated from the relevant ecological context. On the contrary, they are intended as a first step to inform the construction of ecological and epidemiological models where *Wolbachia*, other symbionts, or interventions that modify host susceptibility to infection, are introduced to induce desired transitions in populations. The introduction of *Wolbachia* into *Aedes aegypti* and other arthropod vectors is being considered as a promising strategy to control dengue and other infectious diseases of humans (see [35] and references therein). The inference frameworks presented can be readily adapted to provide accurate quantification of *Wolbachia*-induced protection and integrated in population models of public health importance.

The challenge of considering the time dependence of processes leading to observable ecotoxicity responses has also been addressed in toxicology where the so-called General Unified Model of Survival (GUTS) has been proposed [18]. These models simulate the time-course of external and internal processes leading to toxic effects on organisms to generate an output that can be fitted to mortality over time. While those studies tend prioritize the mechanistic descriptions of the toxicokinetic and toxicodynamic processes that damage the organisms, we have chosen to adopt a phenomenological approach and focus on the inference and interpretation of how susceptibility to infection is distributed in a population.

In epidemiological systems, the baseline transmission intensity is often not directly measurable but indirectly inferred in a model-based manner. Dose-response models, on the other hand, can account for experimentally controlled patterns of exposure [36], [37]. Variation in host susceptibility to pathogens is one component of both classes of systems that mostly influences estimates of intervention impacts [29]. Therefore, building on the methods developed here furthers our potential to accurately evaluate the burden of infectious diseases and design effective interventions.

## Supporting Information

Dataset S1 **Survival data for Wolb^−^ and Wolb^+^ groups of** ***D. melanogaster*** **challenged by various doses of** ***D*** **CV.**
(XLS)

Text S1 **Homogeneous vs heterogeneous model selection.**
(PDF)

## References

[1] Holmes FO (1929) Local lesions in tobacco mosaic. Botanical Gazette 87: 39–65.

[2] Henry JL, Jaikaran ES, Davies JR, Tomlinson AJH, Mason PJ, et al (1966) A study of polio vaccination in infancy: excretion following challenge with live virus by children given killed or living poliovaccine. J Hyg Camb 64: 105–120. 5219018 10.1017/s0022172400040389 PMC2134687

[3] Hornick RB, Music SI, Wenzel R, Cash R, Libonati JP, et al (1971) The Broad Street pump revisited: response of volunteers to ingested cholera vibrios. Bull N Y Acad Med 47: 1181–1191. 5286453 PMC1749960

[4] Ferreira DM, Neill DR, Bangert M, Gritzfeld JF, Green N, et al (2013) Controlled human infection and rechallenge with Streptococcus pneumoniae reveals the protective efficacy of carriage in health adults. Am J Respir Crit Care Med 187: 855–864. 23370916 10.1164/rccm.201212-2277OC PMC3707375

[5] Haas CN, Rose JB, Gerba CP (1999) Quantitative Microbial Risk Assessment. New York: John Wiley & Sons, Inc.

[6] Druett HA (1952) Bacterial invasion. Nature 170: 288. 12993144 10.1038/170288a0

[7] Teunis PF, Havelaar AH (2000) The Beta Poisson dose-response model is not a single-hit model. Risk Anal 20: 513–520. 11051074 10.1111/0272-4332.204048

[8] Kleczkowski A (1950) Interpreting relationships between the concentrations of plant viruses and number of local lesions. J Gen Microbiol 4: 53–69. 15415557 10.1099/00221287-4-1-53

[9] Furumoto WA, Mickey R (1967a) A mathematical model for the infectivity-dilution curve of tobacco mosaic virus: Theoretical consideration. Virology 32: 216–223. 6025875 10.1016/0042-6822(67)90271-1

[10] Furumoto WA, Mickey R (1967b) A mathematical model for the infectivity-dilution curve of tobacco mosaic virus: Experimental tests. Virology 32: 224–233. 6025876 10.1016/0042-6822(67)90272-3

[11] Therneau TM, Grambsch PM (2000) Modeling Survival Data: Extending the Cox Model, Springer-Verlag.

[12] Hougaard P (1986) A class of multivariate failure time distributions. Biometrika 73: 671–678.

[13] Aalen OO (1988) Heterogeneity in survival analysis. Statistics in Medicine 7: 1121–1137. 3201038 10.1002/sim.4780071105

[14] Halloran ME, Longini IM Jr, Struchiner CJ (1996) Estimability and interpretability of vaccine efficacy using frailty mixing models. Am J Epidemiol 144: 83–97. 8659489 10.1093/oxfordjournals.aje.a008858

[15] Longini IM Jr, Halloran ME (1996) A frailty mixture model for estimating vaccine efficacy. Appl Statist 45: 165–173.

[16] Ben-Ami F, Regoes RR, Ebert D (2008) A quantitative test of the relationship between parasite dose and infection probability across different host-parasite combinations. Proc R Soc B 275: 853–859. 10.1098/rspb.2007.1544 PMC2596906 18198145

[17] Baas J, Jager T, Kooijman B (2010) Understanding toxicity as processes in time. Sci Total Environ 408: 3735–3739. 19969324 10.1016/j.scitotenv.2009.10.066

[18] Jager T, Albert C, Preuss TG, Ashauer R (2011) General unified threshold model of survival - a toxicokinetic-toxicodynamic framework for ecotoxicology. Environ Sci Technol 45: 2529–2540. 21366215 10.1021/es103092a

[19] Huang Y, Haas CN (2009) Time-dose-response models for microbial risk assessment. Risk Analysis 29: 648–661. 19187487 10.1111/j.1539-6924.2008.01195.x

[20] Toth DJ, Gundlapalli AV, Schell W, Bulmahn K, Walton TE, et al (2013) Quantitative models of the dose-response and time course of inhalational anthraz in humans. PLOS Pathog 9: e1003555. 24058320 10.1371/journal.ppat.1003555 PMC3744436

[21] Teixeira L, Ferreira A, Ashburner M (2008) The bacterial symbiont Wolbachia induces resistance to RNA viral infections in Drosophila melanogaster. PLOS Biol 6 12: e1000002. 10.1371/journal.pbio.1000002 PMC2605931 19222304

[22] Hedges LM, Brownlie JC, O'Neill SL, Johnson KN (2008) Wolbachia and virus protection in insects. Science 322: 702. 18974344 10.1126/science.1162418

[23] Ryder E, Blows F, Ashburner M, Bautista-Llacer R, Coulson D, et al (2004) The DrosDel collection: a set of P-element insertions for generating custom chromosomal aberrations in Drosophila melanogaster. Genetics 167: 797–813. 15238529 10.1534/genetics.104.026658 PMC1470913

[24] Chrostek E, Marialva MSP, Esteves SS, Weinert LA, Martinez J, et al (2013) Wolbachia veriants indice differential protection to viruses in Drosophila melanogaster: A phenotypic and phylogenomic analysis. PLOS Genet 9: e1003896. 24348259 10.1371/journal.pgen.1003896 PMC3861217

[25] van der Werf W, Hemerik L, Vlak JM, Zwart MP (2011) Heterogeneous host susceptibility enhances prevalence of mixed-genotype micro-parasite infections. PLOS Comput Biol 7: e1002097. 21738463 10.1371/journal.pcbi.1002097 PMC3127814

[26] Patil A, Fonnesbeck CJ (2010) PyMC: Bayesian Stochastic Modelling in Python. J Stat Softw 35: 1. 21603108 PMC3097064

[27] Pessoa D (2014) DISE Dose-Invariant Susceptibility Estimator. Database: Gitgub. https://github.com/dpessoaIGC/Dose-Invariant-Susceptibility-Estimator.

[28] Spiegelhalter DJ, Best NG, Carlin BP, van der Linde A (2002) Bayesian measures of model complexity and fit. J R Statist Soc.B 64: 583–639.

[29] Gomes MGM, Lipsitch M, Wargo AR, Kurath G, Rebelo C, et al (2014) A missing dimension in measures of vaccination impacts. PLOS Pathog 10 3: e1003849. 24603721 10.1371/journal.ppat.1003849 PMC3946326

[30] Souto-Maior C, Lopes JS, Gjini E, Struchiner CJ, Teixeira L, et al (2014) Heterogeneity in symbiotic effects facilitates Wolbachia establishment in insect populations. Theor Ecol (in press).

[31] Osborne SE, Leong YS, O'Neill SL, Johnson KN (2009) Variation in antiviral protection mediated by different Wolbachia strains in Drosophila simulans. PLOS Pathog 5 11: e1000656. 19911047 10.1371/journal.ppat.1000656 PMC2768908

[32] Frentiu F, Robenson J, Young P (2010) Wolbachia-mediated resistance to dengue virus infection and death at the cellular level. PLOS One 5 10: e13398. 20976219 10.1371/journal.pone.0013398 PMC2955527

[33] Lu P, Bian G, Pan X, Xi Z (2012) Wolbachia induces density-dependent inhibition to dengue virus in mosquito cells. PLOS Negl Trop Dis 6 7: e1754. 22848774 10.1371/journal.pntd.0001754 PMC3404113

[34] Osborne SE, Iturbe-Ormaetxe I, Brownlie JC, O'Neill SL, Johnson KN (2012) Antiviral protection and the importance of Wolbachia density and tissue tropism in Drosophila simulans. Appl Environ Microbiol 78: 6922–6929. 22843518 10.1128/AEM.01727-12 PMC3457512

[35] Iturbe-Ormaetxe I, Walker T, O'Neill SL (2011) Wolbachia and the biological control of mosquito-borne disease. EMBO Rep 12: 508–518. 21546911 10.1038/embor.2011.84 PMC3128286

[36] Pujol JM, Eisenberg JE, Haas CN, Koopman JS (2009) The effect of ongoing exposure dynamics in dose response relationships. PLOS Comput Biol 5: e1000399. 19503605 10.1371/journal.pcbi.1000399 PMC2685010

[37] Mayer BT, Koopman JS, Ionides EL, Pujol JM, Eisenberg JN (2011) A dynamics dose-response model to account for exposure patterns in risk assessment: a case study in inhalation anthrax. J R Soc Interface 8: 506–517. 21068030 10.1098/rsif.2010.0491 PMC3061128

