# Supplementary material for: Unveiling Time in Dose-Response Models to Infer Host Susceptibility to Pathogens
Source: PLoS Comput Biol. 2014 Aug 14;10(8):e1003773. doi: 10.1371/journal.pcbi.1003773 (PMC4133050; doi:10.1371/journal.pcbi.1003773)
Supplement: Text S1 — Homogeneous vs heterogeneous model selection. (PDF) [file pcbi.1003773.s002.pdf]

Text S1 – Homogeneous vs heterogeneous model selection

The time-dependent dose-response model constructed in Methods was fitted to the Wolb$^-$ and Wolb$^+$ flies, considering independence of all dose-response parameters, while old-age and baseline mortality (m_0, s_0, κ) as well as challenge imperfection (ε) were assumed constant through the entire dataset. The estimated values for each model and group are presented in Table S1 while the fits are displayed in Figures S1 and S2.

The deviance information criterion (DIC) selected the homogeneous model for Wolb$^-$ (1802 for homogeneous and 1904 for the heterogeneous model) and the heterogeneous for Wolb$^+$ (2680 for homogeneous and 944 for heterogeneous).

Table S1. Estimated parameters from survival curves, comparing the homogeneous and heterogeneous dose-response models.

Mortality data	Model	Parameter	Median	95% HPD
Wolb$^-$ & Wolb$^+$		m_0	114.87	[113.78, 115.83]
		s_0	144.57	[125.20, 163.43]
		ε	2.16 10^{-3}	[1.77 10^{-3}, 2.52 10^{-3}]
		κ	3.01 10^{-3}	[1.24 10^{-3}, 4.75 10^{-3}]
Wolb$^-$		m_1^-	9.31	[9.13, 9.48]
		s_1^-	5.35	[5.01, 5.74]
	homogeneous	p_{hom}^-	1.62 10^{-6}	[8.91 10^{-7}, 2.50 10^{-6}]
	heterogeneous	p_{het}^-	8.36 10^{-6}	[1.22 10^{-6}, 3.63 10^{-5}]
		a_{het}^-	1.51	[0.48, 8.37]
		b_{het}^-	6.43	[1.46, 10]
Wolb$^+$		m_1^+	9.31	[9.13, 9.48]
		s_1^+	36.34	[29.34, 44.52]
	homogeneous	p_{hom}^+	1.50 10^{-7}	[8.39 10^{-8}, 2.35 10^{-7}]
	heterogeneous	p_{het}^+	9.43 10^{-6}	[8.43 10^{-7}, 4.05 10^{-5}]
		a_{het}^+	0.40	[0.22, 0.62]
		b_{het}^+	7.17	[2.27, 10]

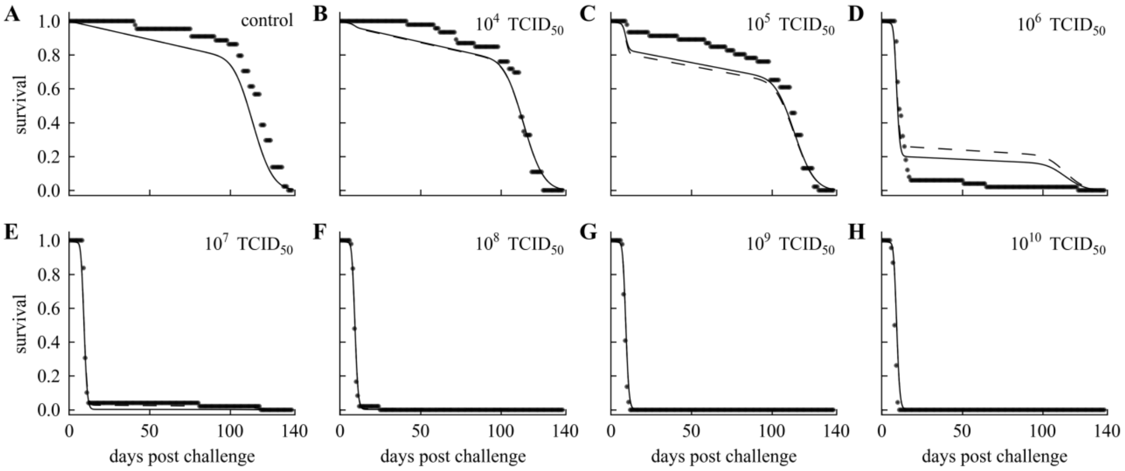

Figure S1. Fit of homogeneous and heterogeneous dose-response models combined with survival over time to Wolb$^-$ survival data. Solid lines are for the homogeneous model, while dashed lines represent the heterogeneous model.

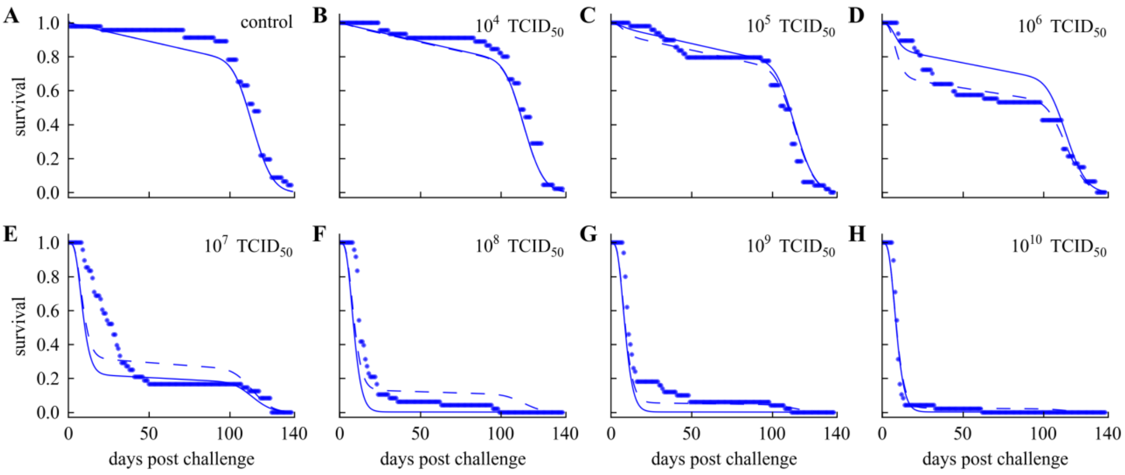

Figure S2. Fit of homogeneous and heterogeneous dose-response models combined with survival over time to Wolb$^+$ survival data. Solid lines are for the homogeneous model, while dashed lines represent the heterogeneous model.
